# Experiences of pain and pain management in advanced disease and serious illness for people from South Asian communities in Leeds and Bradford: a qualitative interview study

**DOI:** 10.1186/s12904-023-01208-2

**Published:** 2023-07-19

**Authors:** Gemma Clarke, Jodie Crooks, Michael I. Bennett, Zarina Mirza, Ruby Bhatti OBE, Wali Nazar, Rahila Mughal, Shenaz Ahmed

**Affiliations:** 1 St Gemma’s Academic Unit of Palliative Care, Division of Primary Care Palliative Care and Public Health, Leeds Institute of Health Sciences, University of Leeds, Level 10, Worsley Building, Leeds, LS2 9NL UK; 2 Policy and Research Team, Marie Curie, London, UK; 3 St Gemma’s Academic Unit of Palliative Care, Division of Primary Care Palliative Care and Public Health, University of Leeds, Leeds, UK; 4 Bradford District Care NHS Foundation Trust, Bradford, UK; 5 Independent Patient and Public Involvement (PPI) Representative, Bradford, UK; 6 Leeds Institute of Health Sciences, University of Leeds, Leeds, UK

**Keywords:** Pain management, Advanced disease, Serious illness, Ethnicity, South Asian communities, Qualitative

## Abstract

**Background:**

Pain is a significant problem for many people with advanced disease or a serious illness. Culture and ethnicity can affect the experience and management of pain. However, there is limited research in South Asian communities in the UK on their experiences of pain. The aim of this study is to explore the experiences and attitudes of patients and family carers from South Asian communities about pain and its management within advanced disease or serious illness.

**Methods:**

Qualitative thematic analysis based on descriptive phenomenology (Sundler et al. 2019). Qualitative semi-structured interviews with patients or family carers from South Asian communities (N = 15). Interviews were recorded, transcribed and analysed using an inductive approach. Public and Patient Involvement representatives from British South Asian communities were consulted for guidance.

**Results:**

There were five key themes from the interviews: The importance of communication about pain with healthcare professionals; Concerns about taking pain medication; Personal resilience, privacy and self-management; Gender, culture and pain; Home pain management as struggle and frustration.

**Conclusion:**

To improve pain management for people from South Asian communities with advanced disease or a serious illness, there are a number of important issues for healthcare professionals from palliative and primary care services to address. These include: greater awareness around people’s fears and concerns about pain medication; their potential use of alternative pain management strategies; and cultural issues such as resilience, privacy, dignity and gender roles. Effective communication between doctors, patients and family members could be improved by using a ‘cultural humility’ model; providing clear and accessible pain medication information; understanding and taking account of people with both low, and medium levels, of English language proficiency; and improving patient trust. Additionally, improvements to out of hours services could improve pain management for all patients managing their pain at home.

**Supplementary Information:**

The online version contains supplementary material available at 10.1186/s12904-023-01208-2.

## Background

Pain is a significant problem for many people with advanced disease or a serious illness. The prevalence of pain ranges from 11 to 98% across a range of different serious illnesses and advanced diseases in the UK and worldwide [1, 2]. Pain is a multifaceted phenomenon; it can be related to social and cultural factors, as well as neurological and physiological responses [3]. The biopsychosocial model of health recognises the dynamic relationships between the biological, social and psychological aspects of a person’s condition [4]. Because the experience of pain is subjective, it can be impacted by these relationships [5]. Culture and ethnicity can affect the experience and treatment of pain in a number of ways, including; definitions of ‘normal’ and ‘abnormal’, beliefs about causes, coping, decision-making, and health-seeking behaviour [4, 5].

Unlike fixed racial categories, both ethnicity and culture are dynamic processes. Senior and Bhopal (1994; p.327) define ethnicity as; “shared origins or social background; shared culture and traditions that are distinctive, maintained between generations, and lead to a sense of identity and group” [6]; and culture can be defined as, “the totality of socially transmitted behaviour patterns, arts, beliefs, institutions” (Dogra, 2010; p.367) [7]. Individuals and communities shape and reshape their own ethnicity and culture through their actions, although these actions are constrained by social and economic forces [8]. Ahmad (1996; p. 190) defines culture as “a flexible resource for living”, rather than a rigid, constraining concept [9]. In the UK, the South Asian community represents one of the largest social and cultural ethnic groups [10], with people originating from many parts of the Indian subcontinent, speaking various languages, and practicing various religions [11].

### Previous research

Some previous research has examined the palliative care experiences of people from South Asian communities living in the UK. A Scottish study of South Asian patients with advanced disease found limited awareness about hospices, difficulties discussing death, and a lack of culturally appropriate care [12]. Two studies set in East London examined the views of older people from South Asian groups and reported a lack of knowledge and discussion about palliative care, with decision-making delegated to family members [13, 14]. One research study explored the experiences of older South Asian people dying in acute hospitals in London and found a high level of mistrust in the care provided, as well as a strong role for families providing ‘a protective shield’ and not letting their loved be alone [15]. A further study examining both patients’ and families’ views and experiences of palliative care in Luton found services were valued, but highlighted the need for improved communication [16]. However, there is a paucity of research on pain and pain management for those with advanced disease or serious life-limiting illnesses within these communities.

Outside of the palliative care context, evidence indicates that people from South Asian communities in the UK are more likely to report chronic and musculoskeletal pain, as compared to other ethnic groups [17–20]. Suggested reasons for this include; lower levels of vitamin D [20], and differences in health-seeking behaviour such as more frequent visits to primary care services [18, 19]. Other evidence indicates that, despite the higher levels of reported pain, pain may still be under-reported by people from South Asian communities due to reasons of cultural stoicism and masculinity [21, 22].

The aim of this study is to explore the experiences and attitudes of patients and family carers from South Asian communities about pain and its management within advanced disease or serious illness.

## Methods

Semi-structured qualitative interviews in-person and via telephone. The theoretical and methodological approach combines descriptive phenomenology with an adapted version of thematic analysis [23]. The research question is: What are the experiences and attitudes of patients and family carers from South Asian communities about pain and its management within advanced disease or serious illness?

### Ethical approval

This research study received UK HRA (Health Research Authority) Greater Manchester Central Research Ethics Committee approval, and HRA overall approval on 08/08/19.

### Theoretical and methodological framework

This study follows the framework presented in Sundler et al. [23], which combines the theoretical principles from descriptive phenomenology, with an adapted style of thematic analysis. The descriptive phenomenology used within Sundler et al. [23] is drawn from the work of Husserl and the concept of the ‘lifeworld’ [24]. The ‘lifeworld’ refers to the world of lived experience, incorporating the ways in which phenomena (events, objects, or emotions) appear within an individual’s conscious perspective [24]. Thematic analysis is a method for analysing qualitative data that entails searching a dataset to identify and report on repeated patterns [25]. Sundler et al.[23] link the methodological principles from thematic analysis, with the philosophical underpinnings of descriptive phenomenology. For example, they link the idea of keeping an open mind when searching for meaning in data, to the sensitive open stance required for understanding lived experience. Drawing upon the phenomenological approaches of Dahlberg et al., they argue this requires questioning the understanding of the data [26], while being observant, attentive and sensitive to the expression of experiences [27]. This approach was chosen because it focuses on understanding the lived experiences of the participants which is useful for exploring pain and pain management.

### Participants and sampling

A purposive sample of N = 30 participants was initially designed drawing upon similar studies and using the ‘Information Power’ matrix from Malterud [28]. However, because of time and resource constraints due to the COVID-19 pandemic, the study closed upon N = 15 participants. (See the limitations section below).

Eligibility criteria:

- (1) Any person with self-identified South Asian ethnicity, including African-born South Asian.
- (2) Any person with advanced disease or serious illness experiencing pain, or family carer of a person with this condition. ‘Advanced disease’ was defined as any life-limiting condition that is unlikely to be cured by treatment [29]. A ‘serious illness’ was defined as any health condition that carries a high or medium/high risk of mortality and commonly affects a patient for several years or longer; and either negatively impacts a person's daily function or quality of life, or excessively strains their caregivers [30].
- (3) Aged eighteen years or older.
- (4) Capacity to give informed consent for an interview.
- (5) Speaker of English or Urdu or Punjabi.

To explore any differences in experiences, participants were sought from two healthcare service and condition-related groups: those with advanced disease utilising specialist palliative care services, including hospice and community, and those with a serious illness or advanced disease being managed by primary care.

### Recruitment

Participants were recruited through specialist palliative and primary care services across Leeds and Bradford. Local services recruited potential participants in two ways: (1) Healthcare staff screened caseloads for potential participants. A local site clinician then approached eligible people directly, explained the study, answered any questions, and gave them a study information sheet to consider. (2) An optional poster advertisement was given to recruitment sites to be placed in public areas if they chose to, or the poster could be used as an ‘advertisement flyer’ for circulation by the site, for example by printing it out and placing it in public areas. Potential participants could use the information on the poster to contact the study team directly if they wanted to take part. Advertisement respondents were asked initial screening questions, and if they were eligible; the research team explained the study, answered any questions, and sent out a study information sheet to them.

After at least 48 h consideration time, the research team got back in touch with potential participants. If they wanted to take part, an interview was scheduled. Informed consent (written or oral) was taken by a member of the research team at the time of interview.

### Data collection

The semi-structured interview guide included questions and prompts on: illness/condition; service use; relationships with healthcare professionals; speaking about pain; medications and other treatments; experiences of friends and family; experiences in other countries; strong medications. (See supplementary materials). Consenting participants were interviewed in-person between October 2019 and February 2020 (n = 5), and via telephone due to the COVID-19 pandemic between October 2020 and May 2021 (n = 10). The interviews were designed to last 30–45 min. The length of the interviews ranged from 15 min to two hours, however the majority of interviews were between 30 to 45 min in length. Interviews were conducted in English by GC (female, English, qualitative researcher). Informed consent was either written or audio recorded. All interviews were audio recorded, with the exception of one participant who chose not to be audio recorded. Audio-recorded interviews were confidentially transcribed verbatim by an experienced transcriber at the University of Leeds. For the one interview that was not audio recorded, a local research nurse transcribed hand-written notes with as much detail as possible.

### Data analysis

The analysis process from Sundler et al. is data driven and involves a reflective process designed to illuminate meaning [23]. Researchers’ subjectivity is an important analytic resource in reflexive thematic analysis for illuminating meaning [31]. GC and JC (White British ethnicity) are experienced qualitative researchers in pain management and undertook the initial readings and explorations of the transcripts, using NVivo to manage the data. Themes were initially explored and patterns began to emerge, which were organised for presentation to the rest of the team for review and feedback (Stages 1 and 2). The themes were then discussed and refined in an iterative process in consultation with SA an experienced British Pakistani qualitative researcher; and RB a PPI representative of South Asian ethnicity with experience as a family carer (Stage 3). Developed themes were reviewed for further cultural nuances and local Leeds and Bradford perspectives with: ZM a research nurse of South Asian ethnicity; and WN and RM former local ethnic liaison officers of South Asian ethnicity (Stage 3).

**Fig. 1:**
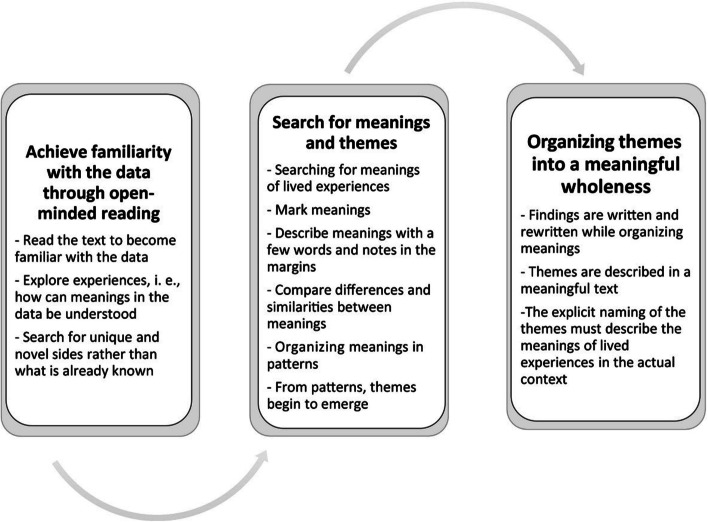
Data analysis process from Sundler et al. 2019

The process of data analysis is drawn from Sundler et al. [23], (see Fig. 1 below). Stage 1: Achieving familiarity with the data through open-minded reading (GC and JC). Stage 2: Search for meaning and themes (GC and JC). Stage 3: Organising themes into a meaningful wholeness (GC, JC, SA, RB, ZM, WN, RM).

### Patient and Public Involvement (PPI)

Patient-facing documents (information sheet, consent form, reply slips) were reviewed by the Research Advisory Group and the Cardiovascular Disease PPI group, both at St James’s Hospital in Leeds. During the first round of data collection, a study specific PPI group was organised in Bradford. Four South Asian community patient representatives attended for oversight and review of the study. Emerging topics, minor refinements to the interview guide and recruitment procedures were discussed. No confidential information or research findings were shared. At the analysis stage, RB who is a lay member of the research team member was involved in the process of analysis. (See description of analysis above).

## Results

### Participant demographics

Fifteen interviews were undertaken. See Table 1 for participants’ demographic information. Participants coded ‘PA’ are from patient participants, and those coded ‘FC’ are from family carer participants.

**Table 1:** Cross-tabulation of participant demographics (N = 15)

Participant type	Participant gender	Patient main condition^a^	Patient age range (years)	Patient religion	Specialist palliative or primary care	Code
Patient	Female	Heart and arthritis	61–75	Muslim	Primary	PA01
Family carer	Female	*Relative 1:* Dementia and arthritis *Relative 2:* Frailty	*Relative 1:* 76–90 *Relative 2:* 91 +	Sikh	Primary	FC01
Family carer	Female	COPD, heart and brain haemorrhage	76–90	Muslim	Primary	FC02
Patient	Female	Arthritis, pain and autoimmune condition	46–60	Muslim	Primary	PA03
Family carer	Male	Cancer	61–75	Muslim	Palliative	FC05
Patient	Male	Advanced skin and tissue condition	18–30	Muslim	Palliative	PA08
Patient	Male	Cancer	61–75	Muslim	Palliative	PA07
Family carer	Female	Cancer	75–80	Sikh	Palliative	FC04
Patient	Male	Heart and genetic muscular condition	31–45	Muslim	Primary	PA06
Patient	Female	Arthritis and pain condition	31–45	Muslim	Primary	PA05
Patient	Female	Cancer	18–30	Muslim	Palliative	PA04
Family carer	Male	Kidney and heart disease	76–90	Muslim	Primary	FC03
Patient	Female	Cancer	31–45	Muslim	Palliative	PA10
Patient	Male	Cancer	61–75	Sikh	Palliative	PA09
Patient	Female	Heart and pain condition	71–80^b^	Muslim	Primary	PA02

^a^Exact names of rare conditions/diseases have been generalised for anonymity purposes

^b^Different age scale asked at interview

#### Themes

The analysis identified five interrelated key themes: (1) The importance of communication about pain with healthcare professionals; (2) Concerns about taking pain medication; (3) Personal resilience, privacy and self-management; (4) Gender, culture and pain. (5) Home pain management as struggle, burden and frustration.

Theme 1: The importance of communication about pain with healthcare professionals.

Communication about pain with healthcare professionals was a key theme throughout the interviews; some participants reported good communication, and others reported poor experiences. Good communication with healthcare professionals increased the participants’ understanding about their pain, but it was also valued for its own sake. Those who spoke about good communication were happier with their care overall. Good communication was experienced as: having rapport, being given individual attention, being understood and feeling ‘heard’, having their suggestions taken seriously, and speaking with healthcare professionals who were knowledgeable and who gave them time.

*“They* [healthcare professionals] *get where I’m coming from. I think so far I’ve, whoever I’ve been involved with myself, where she’s concerned, we’ve had a good understanding and they work well with us, and they’ve looked at our suggestions as well as their own, and taken that on board, and obviously worked together to get to where she is today I think.”* (FC02 Primary)

However, some participants struggled with poor communication experiences, including: having to repeatedly re-explain their pain, not feeling ‘heard’ when they reported pain, or feeling like they were not being believed about the severity of their pain and other symptoms. This could sometimes lead to a misdiagnosis. A participant with advanced cancer, who was diagnosed late, explained the frustration of first being diagnosed with sciatica:

*“*(Dr name) *said it’s sciatica. Cos I told him, I said look, it’s hurting me here… When I had this ‘so called’ sciatica pain, and this doctor... I went to him, and at that time I had lost my appetite, you know, I was losing a lot of weight. So I went and told him, doctor I don’t think it’s sciatica, it’s got to be something else, why am I losing weight, why am I not eating?”* (PA07 Palliative)

The result of poor communication about pain and other symptoms was often a delay in getting access to treatment:

*“We were getting fed up with the GP...they would say ‘well I just saw you last week, I can’t do anything, it’s just pain.’ So I did go back a month later to complain what they’d been delaying, why didn’t they refer him properly, but basically they, they didn’t refer him on time because they didn’t think there was anything serious.”* (FC05 Palliative)

Difficulties with communication could be compounded by issues with English language proficiency. All of the participants spoke English, however some expressed concerns for older family members who were not native English speakers. This was not just about those who could not speak English at all, but also about those with lower proficiency. Older people from South Asian communities could present with conversational English, so a healthcare professional would be unaware of any difficulties, however they could still struggle with understanding technical medical language about their condition or their medication. One participant described how difficulties with English language proficiency and understanding technical medical language could be particularly pernicious issues, because they could be compounded by feeling tired due to experiencing pain, or by lacking the confidence to speak up and ask for clarification:

“*Mum and Dad’s English is good, like they can have conversation, but I think sometimes you know, when things get a bit technical, and there is that technically language there, it kind of baffles them, and they kind of go quiet. Especially my dad at the minute because of what he’s, when you’re in pain, you can’t be bothered; you just think, do you know what, leave me alone. And I think for my mum, it’s like oh,* [quieter voice] *‘I didn’t quite understand that’.*” (FC04 Palliative)

Participants who were younger family carers described how they assumed responsibility for their older relatives’ care, and often played a key role in communicating with healthcare professionals. Frequently family members helped as interpreters. Even for relatives who did speak English, family members played a key role in communicating with healthcare staff by making and attending appointments to speak with doctors.

*“I mean even today I’m going to have a rant at one of them* [healthcare staff] *because she’s supposed to have this appointment months ago.”* (FC03 Primary)

However, one participant described how these family dynamics could risk misinterpretation about the patient’s pain experience.

*“He’s, you know, he’s ok with English. His sister couldn’t speak a word of English and so how was she able to explain things to anybody, she would explain to her daughter or her son, and then how much they interpret or mis-interpret is another problem.”* (FC05 Palliative)

Theme 2: Concerns about taking pain medication.

Most of the participants expressed some concerns and fears about pain medication. There were a range of unaddressed concerns about their pain management; from worries that pain medication was not working for their loved one, to concerns about their medication regimens, to apprehensions about the quantities and strength of painkillers being taken:

*“Being on so much* [medication]… *when the doctor suggested to me Oramorph, I said to him, are you feeling alright? I says, you’ve put me on so much medication as it is*.” (PA05 Primary)

The reasons behind these concerns were in two categories: Fears and concerns related to side-effects of pain medication, and hesitancy caused by a perception of pain medication as ‘unnatural’. Firstly, some participants had worries about the side-effects they had already experienced, and some had fears about potentially experiencing more or much worse side-effects.

*“I know that he would probably think that some of it may have issues on his bowels… And that’s one thing that he probably would put him off having anything [pain medication] unless he absolutely had to.”* (FC01 Primary)

*“I don’t take any pain killers whatsoever...Because of the side effects...Just very bad. I don’t like pain killers.”* (PA08 Palliative)

Due to fears about experiencing side effects, some people did not adhere to their medication regimen, instead they only took painkillers when they felt it was absolutely necessary:

*“Yeah there is sometimes very* [side effects], *but still I when I have got pain, and I can’t stand it, then I take.” (PA02 Primary)*

Fears about side effects also included more significant fears about hastening death. One person also expressed a fear that morphine could kill someone more quickly.

*My grandma, she was 97 and she just kind of died of old age, but it was over the last 3 days she was given morphine, just because her heart was failing. And they came and gave her morphine but I think you just hear things or people say that, when you’re on morphine it kind of kills you quicker. I don’t know anything on that.* (FC04 Palliative)

Secondly, there was a hesitancy caused by an idea that pain medication was a ‘drug’ and therefore was seen as ‘unnatural’ and demanding on the body. Sometimes this was positioned in opposition to the natural ‘body’ and to good ‘health’.

*“Dad was very reluctant to take morphine, because I think he thinks that it has like a negative impact on your body.. But just trying to imagine his mindset and what he would be thinking… I suppose it is a drug isn’t it, so it’s probably not going to be beneficial in the long run.”* (FC04 Palliative)

“*I feel I’m taking a lot of medication and it’s not good for health.”* (PA10 Palliative).

Other participants spoke about medication as a ‘prescriptive solution’ to pain management, they saw medication as a way for healthcare professionals to brush them off during appointments, without taking the time to understand their wishes or their condition. This links back into the experiences of poor communication with healthcare professionals above.

*“It would be really great for just sort of people to, health professionals to communicate and really try and understand the cause of the pain rather than just give drugs and hope for the best”* (PA04 Palliative)

*“My GPs love prescribing tablets, painkillers, it’s from one tablet to another.. but I do not want any more medication.”* (PA03 Primary)

Theme 3: Personal resilience, privacy and self-management.

Personal resilience and privacy were important for many of the participants. Some participants directly linked resilience and home-based self-treatments to South Asian culture, and to growing up in Pakistan or India:

“*If you are asking in the other country* [Pakistan]… *we actually never tell anyone, we used to tolerate the pain first, and the next thing we go for the home remedies… if it is not controllable only, we would go for a hospital.*” (PA10 Palliative)

One participant directly linked the need for privacy, dignity and resilience when receiving personal care to South Asian culture.

*“They* [healthcare professionals] *can’t understand some of the concepts that the South Asian people have in terms of… for example privacy. When my dad was at home, he wouldn’t want anybody to touch him, in terms of changing his nappies.”* (FC05 Palliative)

Other participants drew internal strength from their faith, and used methods of pain relief with religious significance which they believed to be a blessing:

*“Because like in… in my religion, Islam, we believe that we are being tested. So you got to stay patient.”* (PA08 Palliative)

*“In our community like, you know like as a Muslim, cupping for us is part of our religion, yeah? …Yeah, honestly it’s in the Qur’an… cupping for us is a blessing.”* (PA05 Primary)

Most participants had tried some form of alternative or complementary therapy for their, or their loved one’s pain. Many took a ‘natural self-management’ approach using non-prescribed therapies they had researched themselves. Sourcing therapies such as CBD oil, allowed participants to have some power over their own pain treatment, and gave them a sense of control. Although there were mixed reports of success.

*“That’s* [CBD oil] *just for rubbing. Taking the pain away...That’s me prescribed myself, and that is helping, [it’s] not doctors’ thing.”* (PA07 Palliative)

Participant: *“I’ve even tried her on cannabis oil because I thought you know I’d done a little…”. *Interviewer: *“Yeah, did that work?”. *Participant: *“Well I’m not so sure, I’m not so sure you know I did a little bit of research online and stuff and there are people that swear by it. ” (FC03 Primary)*

The ‘natural self-management’ approach links back to the previous theme of hesitancy and prescribed pain medications being perceived as ‘unnatural’. Some participants preferred treatments they saw as more ‘natural’. One participant spoke about using herbal remedies to avoid taking ‘drugs’ (prescribed pain medications).

*“I’m taking a few supplements now from a herbalist after explaining kind of my situation because I don’t want to stay on the drugs I guess.”* (PA04 Palliative)

Theme 4: Gender, culture and pain.

Participants were asked if they thought that culture affects pain management. Many participants responded strongly to this question and answered in terms of their experience. Some expressed that they felt there was no difference in pain experience by someone’s culture or ethnicity.

*“I don’t think pain, I don’t think [the] human body, one human body is different to another in terms of culture or language or ethnicity... How the pain comes with this illness, it’s not going to pick on a racial basis, you know; if you’re White you get more pain, if you’re Black you get less pain, it won’t happen like that will it?”* (FC05 Palliative)

*“Pain’s pain no matter what culture.”* (FC03 Primary).

However other participants felt there was a significant connection between pain and culture. The majority of these participants were women who described gendered differences, such as women’s duty to tolerate pain and continue to perform family duties.

*“I think in Asian families, the women are expected to just get on with it, carry on with it no matter what and that’s what over the years I have done as well.”* (PA03 Primary)

*“It is very cultural for women, you know, to not moan about anything.”* (PA01 Primary)

Another gender issue raised by both male and female participants, was that men were less able to open up and discuss their pain; or were more likely to go straight to the doctor, without telling their family. One male participant discussed how he did not want to worry his wife and family, but felt he could talk more freely to a doctor (PA09 Palliative, written notes).

*“Maybe you know, kind of being from an Asian family, being a man, kind of being strong, I don’t know if it’s a combination of stuff, that he just feels that he can’t open up, and talk about the way he’s feeling.”* (FC04 Palliative)

*“My mum would quite happily try and deal with it [pain] herself and not bother anybody as in going to the GP unless she had to...Whereas my dad is the opposite.”* (FC01 Primary)

Theme 5: Home pain management as struggle, burden and frustration.

One issue that was underlying all the other themes was the struggle and frustration people felt while managing their pain at home in the community. This was because they struggled to access medication and appointments. Home pain management issues were often experienced at ‘out of hours’ times, such as evenings or weekends. Difficulties with obtaining GP appointments were constant, while other access issues were occasional or intermittent but ongoing. Those in an in-patient setting, or those who had previously experienced in-patient care, felt their pain was managed well in that setting. There was no clear-cut divide between those receiving specialist palliative care services and those receiving primary care only for pain management. Rather, the division was between those receiving inpatient care; and those being managed at home.

Not being able to access appointments or medications on time had a significant emotional impact on participants. They felt they had to continually struggle and ‘push back’ to get pain relief for themselves, or for their loved one. This was an extra burden for participants, and compounded the issues in the previous themes.

*“I’ve had trouble with getting medicine on time….. It’s lack of communication with the pharmacy and doctors. Sometimes, it runs out quicker, and then doctors send out emergency prescriptions and then…weeks go …sometimes* [it’s] *order*[ed] *too late, and then it comes late.”* (PA08 Palliative)

*“Yeah I’ve, it’s a constant chase, even with the GP, I mean they’re lovely people, I’m probably making them sound like they’re horrible but I am constantly having to wrestle with them. You know it took me nearly 2 months, 3 months to get the matron out and that was me kicking off with the doctors”* (FC03 Primary)

Challenges in obtaining their medication left many participants feeling frustrated and burdened.

*“It was just such a big faff just to get my medication...But apparently because it was morphine, this was the route they had to go through”* (PA04 Palliative)

“*They’re purposely doing all this,* [delaying medications] *I think they like* [it] *when we’re swearing at them.”* (PA07 Palliative)

## Discussion

This qualitative interview study contributes to the understanding of pain and pain management for people with advanced disease or a serious illness from South Asian communities living in the UK. The findings revealed five key themes: the importance of good communication; unaddressed concerns about pain medications; South Asian culture and the relationship to personal resilience, privacy, dignity, and self-management; gendered differences and culture in pain management; and those receiving care at home struggled to manage their pain and get access to medications and appointments.

The US Joint Commission defined three key components to addressing effective communication in healthcare for people who do not speak English: i. language barriers, ii. cultural competency and iii. healthcare literacy [32]. This framework is informative for understanding and interpreting the themes from this study.

*i*. *Language barriers.*

One of the key themes from this study was the importance of good communication with healthcare professionals. The findings revealed that language barriers can occur for people with both low levels, and medium levels, of English proficiency. Importantly, this could mean that healthcare professionals are unaware of the language-related needs of some patients during interactions, particularly if they have a medium level of English language proficiency. Other research has shown that even for bilingual people who are fluent in two languages with high levels of proficiency, they may prefer to communicate in their first language during times of stress and ill health [33]. A further issue highlighted by discussions with PPI representatives was the difficulty that some older adults from South Asian communities have difficulties with general reading and reading English language. This means they can struggle to read the labels and instructions on their pain medication. This is related to the key theme of concerns about medications. A study of bilingual prescribing in community pharmacies in the UK found improved patient understanding and less need for patients to ask a third party for assistance with their medication [34]. This is an important area for future policy initiatives in both palliative and primary care in terms of the availability and training of medical interpreters; assessing patient and carer’s language needs, and the importance of clear written information in languages other than English.

A subtheme within the theme of communication was the role of family dynamics within pain communication for South Asian patients and families, and the potential for these dynamics to lead to misunderstandings. Due to the invisible nature of pain, individuals can struggle to communicate about it with healthcare professionals, who may show less empathy to patients when they cannot see physical signs or symptoms of pain and disease [35]. These conversations can be even more complex when families and wider social networks are involved, and the person experiencing the pain has low English language proficiency. There is a paucity of research into pain communication and family dynamics in all communities. However, the small amount of research conducted on this topic has illustrated the importance of understanding these complex dynamics. One study found that the ability of older people to communicate about pain within family networks was both a resource (by allowing people to get help or by strengthening interpersonal relations), and a challenge (by threatening their autonomy, social relations or self-esteem) [36]. Another scoping review of pain communication in families when parents were experiencing pain, found it was dependent on a number of factors including, the skills that members possess which they perform both individually and together [37]. The lack of evidence on pain expression and South Asian family dynamics within palliative care, warrants specific attention because the experience of pain is subjective, invisible to others, and open to different cultural, social and psychological influences [5]. Understanding the complex intra- and inter-personal dynamics of expressing pain between families and patients is important for healthcare professionals and medical interpreters to work better with patients and their families.

*ii. Cultural competency.*

Two of the key themes were related to South Asian culture, these were ‘personal resilience, privacy and self-management’ and ‘gender, culture and pain’. These two themes illustrated that greater cultural understanding is required around: privacy for receiving personal care; resilience and expressing pain; and gendered differences. Additionally, the subtheme of ‘natural self-management of pain’ showed that patients’ potential use of a variety of alternative pain treatments can be related to South Asian culture. This is an important issue for healthcare professionals to consider and discuss with patients. These findings align with other research into the importance of privacy, masculinity and alternative therapies in healthcare for patients from South Asian communities [21, 38].

Although greater cultural understanding is required around these issues, the findings presented in this paper should not be understood as prescriptive, they are part of an ongoing conversation about improving pain management for people from South Asian communities. Culture is flexible and people have individual responses to cultural norms [9]. The ‘cultural humility’ model may be useful for understanding these findings, and applying them in practice. It places the patient as expert, and requires that cultural knowledge is not reified as a difference, but rather utilised as part of an ongoing conversation between the patient, the family and the healthcare provider [39]. Cultural humility describes a life-long, reflective process (rather than being about achieving an end-goal) that recognises power imbalances between health professionals, researchers and patients [40]. For working cross-culturally in practice, Robinson et al. created a model of cultural humility for healthcare leaders grounded in 5 Rs: Reflection, Respect, Regard, Relevance, Resiliency (see Table 2) [41]. The goal of the 5 Rs is to overcome inertia, creating greater equality and inclusion in diverse healthcare settings [41].

**Table 2:** The 5 Rs of Cultural Humility [41]

Reflection	**Aim:** One will approach every encounter with humility and understanding that there is always something to learn from everyone **Ask:** What did I learn from each person in that encounter?
Respect	**Aim:** One will treat every person with the utmost respect and strive to preserve dignity and respect **Ask:** Did I treat everyone involved in that encounter respectfully?
Regard	**Aim:** One will hold every person in their highest regard while being aware of and not allowing unconscious biases to interfere in any interactions **Ask:** Did unconscious biases drive this interaction?
Relevance	**Aim:** One will expect cultural humility to be relevant and apply this practice to every encounter **Ask**: How was cultural humility relevant in this interaction?
Resiliency	**Aim:** One will embody the practice of cultural humility to enhance personal resilience and global compassion **Ask:** How was my personal resiliency affected by this interaction?

Source: Robinson et al.[41]

*iii.* *Health literacy.*

The theme of ‘concerns about medication’ showed that patients had fears about pain medication being ‘unnatural’, and thus bad for their health or body. This could be the result of low health literacy and a lack of understanding about their condition or their medication. For those participants with advanced disease, it may also represent a lack of understanding or denial about their or their loved one’s prognosis, and a misunderstanding about the aims of palliative treatment. Other research has shown that lower levels of health literacy can affect patients’ self-management of pain [42–44], and may impact upon pain intensity [45]. This is thought to be due to the impact of lower health literacy on a number of health behaviours which are important for managing pain, such as; adherence to treatment and medication [46, 47], and communication and engagement with healthcare professionals [48–50]. These factors have also been highlighted as important by the findings from this study as illustrated within the communication and concerns about medication themes.

Health literacy has been shown to moderate the relationships between health inequalities, health outcomes and socially disadvantaged groups within society, for example racial inequalities [51–53], and socioeconomic status [54]. However, the relationships between health inequalities, health literacy and social disadvantage in society are complex. Health literacy is tightly linked to a number of multilevel factors which are related to the unequal distribution of resources in society, such as access to education, low income, poor housing, disability, and being part of a vulnerable group [50]. In this way, health literacy may be considered more descriptive, than interventional. Moreover, the concept of health literacy places the onus upon the patient, who may already be part of a marginalised group, rather than placing the responsibility on the healthcare system. Friesen and Gligorov argue that the failure to consider racial inequalities in pain treatment as a causal factor in pain outcomes, is an example of discrimination and White ignorance [55]. Healthcare interventions need to be delivered in a culturally sensitive way for each group, before comparing levels of health literacy, and describing those from minoritised groups as in deficit.

There has been little research into the health literacy for people from South Asian communities in the UK. Further research and policy initiatives are required to improve the information about pain presented to patients in terms of understandability. Working with patient representatives is crucial to reduce medical jargon and present information in a clear readable way.

### Patient trust

A related concept to effective communication is patient trust. The theme ‘concerns about medication’ showed that participants had fears about pain medication as ‘unnatural’ and harmful. This may be the result of a lack of trust in the healthcare system and healthcare professionals, rather than simply the result of poor communication or a lack of knowledge as described above. The communication theme also revealed that some of the participants felt they were disbelieved when they reported their pain. This finding on disbelief aligns with a body of literature from the US which has found that people from minoritised racial groups are more likely to have their pain underestimated and disbelieved, and less likely to receive analgesics, than people from the White group [5, 56]. This study did not compare the experiences of South Asian patients and family carers with any other ethnic group, so similar claims cannot be made, however it is an area for further research in the UK.

Effective communication is one way to improve patient trust at the individual-level [57]. Communication should be two-way, to allow patients to discuss their concerns. However, wider societal changes to reduce inequalities in healthcare, reduce racism and discrimination, and increase representation are also required over the longer term to improve trust.

### Struggle of pain management at home

Another key finding was the struggle and frustration people felt while having their pain managed at home, particularly during ‘out of hours’ periods (evenings, night times and weekends). This is important for understanding and contextualising the findings for two reasons. Firstly, it was the underlying experience for most of those managing their pain at home, struggling to get access to pain medication and appointments was an extra burden which compounded other issues and may have exacerbated their experience of pain. Secondly, evidence shows that people from diverse and minoritised ethnic groups do not have the same access to hospice services, and are less likely to use hospice inpatient services, compared to those from White groups [58], which means that people from South Asian communities may be more likely to have their pain managed by primary care at home, and are thus disproportionately affected by these issues. Difficulties with obtaining appointments and medications out of hours may not be unique to people from South Asian communities. Research has shown that access to primary and palliative care services during out of hours periods is challenging for many people, with patients facing issues such as unsatisfactory symptom management, insufficient consideration of personal preferences, and unnecessary ambulance call outs and hospital admissions [59–62]. There are multifaceted reasons as to why people struggle to access services out of hours and manage their pain at home. Research has shown that those utilising out of hours emergency appointments most frequently include: adults aged over 65 years, women, people from socioeconomically deprived areas, and those with chronic diseases or mental health problems) [63], with up to 40% of appointments going to those from the most socially deprived quintile [64]. Some of the sites recruiting for this study included patients from the most deprived areas, as well as more affluent areas, however socioeconomic information was not collected as part of this study. Further research is required into these complex relationships. Policy initiatives to improve ‘out of hours’ services for medication and appointments would be of benefit to all ethnic and cultural groups in the UK, but could also reduce the disproportionate effect on those with reduced access to hospice, such as people from South Asian communities.

### Limitations

The main limitation was due to the COVID-19 pandemic which meant a smaller number of interviews (N = 15) were undertaken than originally planned (N = 30). This was due to a reduced time period and clinical pressures upon staff. All participants who met the eligibility criteria within this time period were interviewed. A further four people expressed an initial interest and then withdrew. Fifteen in-depth interviews still provided enough data to explore themes, but data saturation was not reached. A larger study with more participants may have revealed more themes and the findings could have been more nuanced.

Another limitation was that all interviews were conducted in English by a female British interviewer. The original protocol planned for some interviews to be undertaken in Urdu or Punjabi by a South Asian interviewer (ZM). RM designed and translated participant information sheets, and ZM facilitated the development of a recruitment strategy for non-English speaking participants. However, these interviews by ZM were ultimately not possible due to time constraints, COVID closures, and two potential Urdu-speaking participants who withdrew. This may have affected the findings of the study. Some researchers argue that ethnically and linguistically matching interviewers and participants can create different, or potentially more accurate, data compared to those who are not matched [65, 66]. This is thought to be the results of increased trust, empathy, rapport building and the construction of a shared understanding [65, 66]. However, they also warn that there is a need to be cautious over partially shared identities, and that other characteristics such age, migration status and gender are also important for building rapport [65]. Further research could include interviewers from different ethnic groups and genders to compare and contrast the findings produced.

## Conclusions

In conclusion, effective communication is crucial for improving pain management for people from South Asian communities. The findings from this study revealed that participants had unaddressed fears and concerns about pain medication, and many felt there were areas of South Asian culture that required greater understanding from healthcare professionals Using a ‘cultural humility’ model for communication could help improve rapport and trust, and more egalitarian conversations between doctors, patients and families on these issues. The findings also suggested that a greater awareness of low and medium English language proficiency is required across healthcare services, even for those with conversational English. Improvements in written materials on pain medication could also help improve pain management and adherence to medication. Working with communities to produce accessible and clear information on pain medication could relieve some patients’ anxieties about pain medication and improve understanding for those with lower health literacy. Patient fears and anxieties about medications, may not only be the result of poor communication and understanding, they may also be related to reduced trust in healthcare. Any improvements made by professionals or services, need to be in the wider context of anti-racist measures taken across the whole healthcare system. Additionally, there have long been concerns about out of hours services in palliative care in terms of lack of access to medication, information transfer and carer support [68]. The findings from this study indicate that improvements on these issues could not only improve pain management for all, but may also reduce the disproportionate impact on people from South Asian communities who are be less likely to access hospice inpatient services [63]and thus more likely to have their pain managed at home.

Research and policy recommendations.

- Co-producing written information and instructions about pain medication and pain related conditions with community and patient representatives, to reduce jargon and increase understandability. Making written information available in the appropriate languages.
- Further investment and policy initiatives to improve the availability and training of medical interpreters, and potentially increase their usage to people with medium English language proficiency.
- More education, cultural understanding and ‘cultural humility’ for healthcare professionals around understanding South Asian culture and pain experiences and management.
- Further research into pain communication, medical interpreters and family dynamics in South Asian communities, including intra- and inter-personal expression and communication of pain.

## Supplementary Information

**Additional file 1.**

## Data Availability

An anonymised version of the qualitative dataset may be available from the lead author upon reasonable request. Please contact the lead author Dr Gemma Clarke via email (g.c.clarke@leeds.ac.uk).

## Abbreviations

FC: Label for quotes from participants who were family carers
GP: General Practitioner of medicine
HRA: Health Research Authority
PA: Label for quotes from participants who were patients
PPI: Patient and Public Involvement
UK: United Kingdom
US: United States of America
