# Supplementary material for: Experiences of pain and pain management in advanced disease and serious illness for people from South Asian communities in Leeds and Bradford: a qualitative interview study
Source: BMC Palliat Care. 2023 Jul 19;22:90. doi: 10.1186/s12904-023-01208-2 (PMC10355000; doi:10.1186/s12904-023-01208-2)
Supplement: Supplementary file 1 — Additional file 1. [file 12904_2023_1208_MOESM1_ESM.docx]

**Pain in advanced disease: South Asians in Leeds and Bradford**

**Interview guide**

***Preamble***

Today I/we would like to speak your experience of pain and pain management. There are no right or wrong answers. I/we just want to hear about your experiences. We/I hope the information you give today can be used to help people with pain management in the future. All the interviews will be anonymised afterwards: all personal details and names will be removed, so you will not be identified.

You do not have to answer all the questions. If you feel uncomfortable you can skip questions and move to the next.

If you would like to withdraw from the study at any point let me/us know. You can do this at any time during the interview today, or up to two months afterwards. There will be no consequences to you and won’t affect your care in any way.

The questions should last around 45 minutes. If you need to take a break at any point or want to reschedule for another day let us know. Do you have any questions before we start?

Prompt => permission to start audio recording

**Topic guide:**

Tell me about your… *(prompts)*

(1) Experiences of pain and your condition;

(2) Pain management and control; *(Medications and other treatments, gender, culture)*

(3) Speaking about and expressing pain symptoms; *(Family and HCPs)*

(4) Seeking help for pain; *(Services in UK and other countries, with HCPs)*

(5) Strong pain management at the end of life. *(If appropriate)*

***Closing interview text.***

Thank you very much for your time today. If you need to get in touch with me afterwards or you think of something you’d like to say my details are on the information sheet.

If you want to speak to someone else, or you’ve found today difficult, there are also details of a Marie Curie helpline.

Would you like to be sent information about the results of this study? [note down response and preferred method of contact]

Thank you again.
